# Clinical picture: SDHD paraganglioma presenting with syncope

**DOI:** 10.1002/ccr3.3630

**Published:** 2020-12-07

**Authors:** David P. LaChance, Thanh D. Hoang, Mohamed K. M. Shakir

**Affiliations:** ^1^ Division of Endocrinology Department of Medicine Walter Reed National Military Medical Center Bethesda MD USA

**Keywords:** paraganglioma, pheochromocytoma, SDHD, syncope

## Abstract

Described is an atypical presentation of a rare condition. It highlights the importance of thorough algorithm of medical and family history, physical examination, appropriate investigations, and perioperative workup and for surgery. The report demonstrates how even such very rare nonsecreting paragangliomas can be secondary to mass effects.

## Graphical abstract

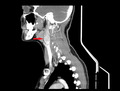

## 1 CLINICAL VIGNETTE

**FIGURE 1:**
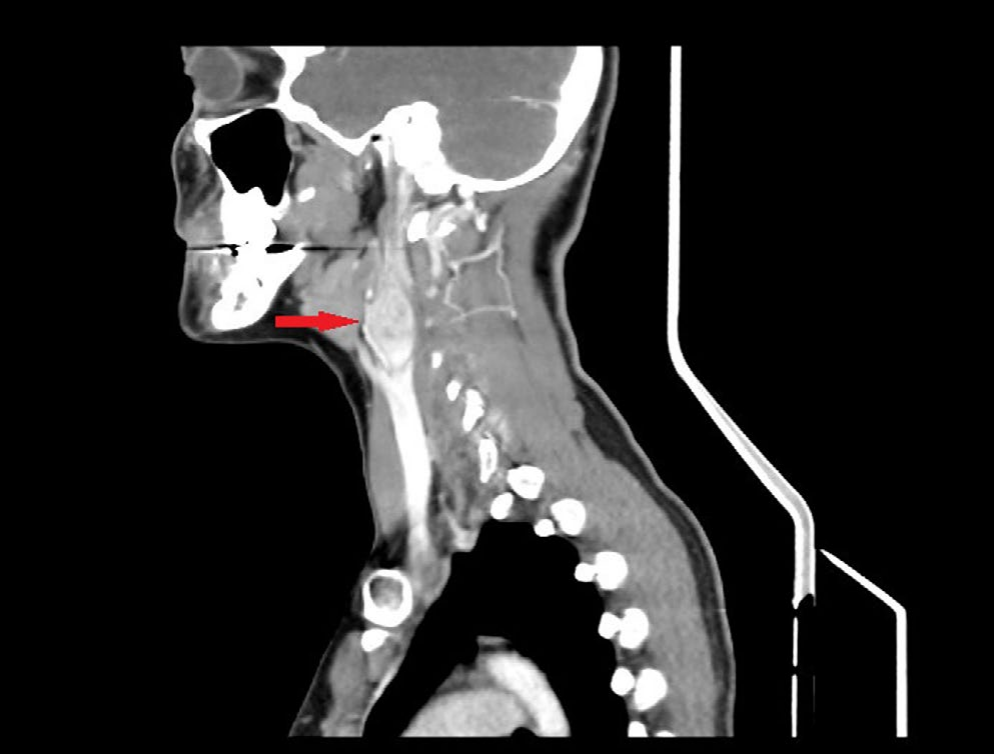
Head/neck CT demonstrating bilateral masses in the carotid bifurcations consistent with carotid body tumors

A 29‐year‐old woman was presented after she lost consciousness and fell while walking. Syncope was preceded by 15 minutes of flushing, nausea, and palpitations. She reported similar episodes in the preceding months. Assessment included normal vital signs, cardiopulmonary auscultation, neck examination, cranial nerve examination, electrocardiogram, echocardiogram, thyroid function, CBC, and CMP. Head/neck CT revealed bilateral carotid body tumors (Figure 1). History revealed a history of bilateral carotid body tumors in her father which were never evaluated. Plasma and urine metanephrines were normal; therefore, alpha‐adrenergic and beta‐adrenergic blockade were not initiated prior to surgical excision given the lack of concern for intra‐operative hypertensive crisis.

**FIGURE 2:**
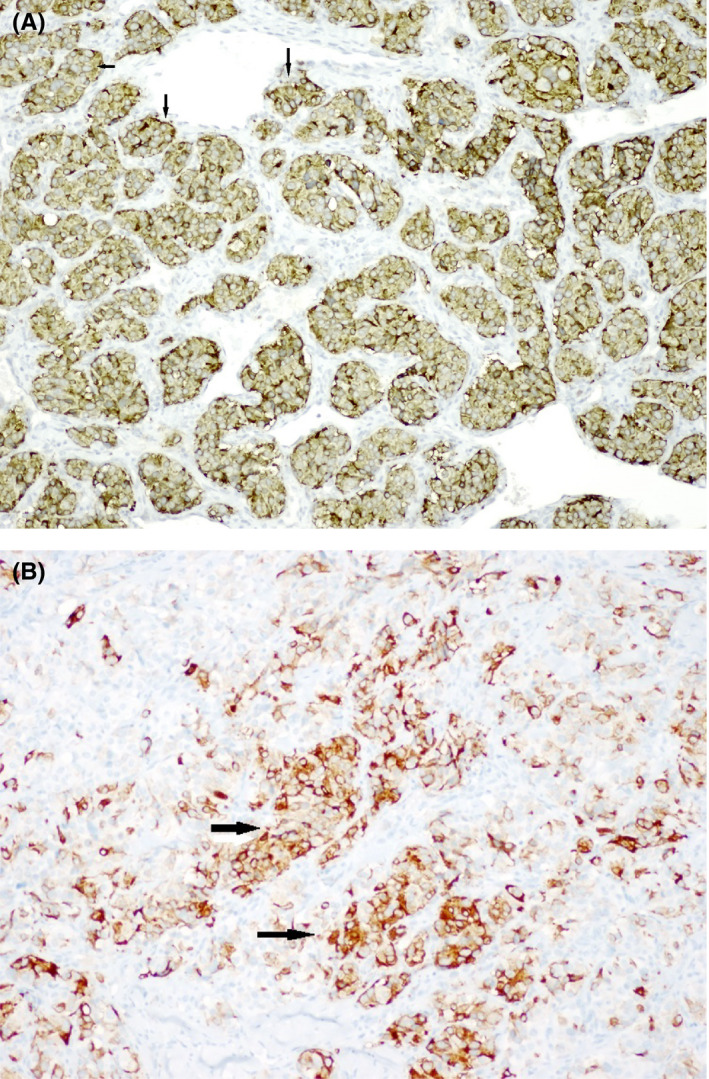
A, Histology of excised paraganglioma showing positive synaptophysin stain. B, Histology of excised paraganglioma showing positive chromogranin stain

The larger carotid body tumor on the left was excised, and pathology revealed a paraganglioma with positive synaptophysin (Figure 2A) and chromogranin (Figure 2B) stains. Genetic testing revealed a succinate dehydrogenase complex subunit D (SDHD) gene mutation. An SDHD gene mutation is associated with hereditary paraganglioma‐pheochromocytoma (PGL/PCC) syndromes which most commonly originate head and neck and form in the carotid body. They usually arise from the autonomic nervous system anywhere from the skull base to the pelvis with an incidence of 1:30 000‐1:100 000.^1^ About 95% of head and neck paragangliomas are nonsecretory.^2^ Symptoms can arise from catecholamine hypersecretion, presenting as hypertension, headaches, diaphoresis, flushing, anxiety, or palpitations, and can be episodic or sustained, or mass effect. Syncope as a presenting symptom is rare and has not been quantified but only reported in case reports.^3^ Hereditary PGL/PCC syndromes should be suspected in any individual with multiple, recurrent, early‐onset (age <45 years), or family history of PGL/PCC, as these syndromes are inherited in an autosomal dominant manner.^2^ Diagnosis is established via genetic testing. There is generally a paternal mode of inheritance and a <5% malignancy risk.^2^ Treatment is resection of the tumor(s) with alpha‐adrenergic blockade pre‐operatively,however, watchful waiting is a reasonable option for nonsecretory, asymptomatic tumors. Patients and first‐degree relatives should be screened annually with biochemical testing and every 2‐3 years with imaging.

## CONFLICT OF INTEREST

None to declare.

## AUTHOR CONTRIBUTIONS

DPL: was an author. TDH and MKMS: were reviewers and editors.

## ETHICAL APPROVAL

This manuscript has been reviewed and approved by the IRB and Public Affairs Office.
